# Characterizing undiagnosed chronic obstructive pulmonary disease: a systematic review and meta-analysis

**DOI:** 10.1186/s12931-018-0731-1

**Published:** 2018-02-07

**Authors:** Kate M. Johnson, Stirling Bryan, Shahzad Ghanbarian, Don D. Sin, Mohsen Sadatsafavi

**Affiliations:** 1 Collaboration for Outcomes Research and Evaluation, Faculty of Pharmaceutical Sciences, University of British Columbia, 2405 Wesbrook Mall, Vancouver, BC V6T 1Z3 Canada; 2 Centre for Clinical Epidemiology and Evaluation, Vancouver Coastal Health Institute, Vancouver, Canada; 3 Centre for Heart Lung Innovation (the James Hogg Research Centre), St. Paul’s Hospital, Vancouver, Canada; 4 Institute for Heart and Lung Health, Department of Medicine, University of British Columbia, Vancouver, Canada

**Keywords:** Delayed diagnosis, Diagnostic errors, Differential diagnosis, Risk factors, Chronic Obstructive Pulmonary Disease, Systematic review, Meta-analysis

## Abstract

**Background:**

A significant proportion of patients with chronic obstructive pulmonary disease (COPD) remain undiagnosed. Characterizing these patients can increase our understanding of the ‘hidden’ burden of COPD and the effectiveness of case detection interventions.

**Methods:**

We conducted a systematic review and meta-analysis to compare patient and disease factors between patients with undiagnosed persistent airflow limitation and those with diagnosed COPD. We searched MEDLINE and EMBASE for observational studies of adult patients meeting accepted spirometric definitions of COPD. We extracted and pooled summary data on the proportion or mean of each risk factor among diagnosed and undiagnosed patients (unadjusted analysis), and coefficients for the adjusted association between risk factors and diagnosis status (adjusted analysis).

**Results:**

Two thousand eighty-three records were identified through database searching and 16 articles were used in the meta-analyses. Diagnosed patients were less likely to have mild (v. moderate to very severe) COPD (odds ratio [OR] 0.30, 95%CI 0.24–0.37, 6 studies) in unadjusted analysis. This association remained significant but its strength was attenuated in the adjusted analysis (OR 0.72, 95%CI 0.58–0.89, 2 studies). Diagnosed patients were more likely to report respiratory symptoms such as wheezing (OR 3.51, 95%CI 2.19–5.63, 3 studies) and phlegm (OR 2.16, 95% CI 1.38–3.38, 3 studies), had more severe dyspnea (mean difference in modified Medical Research Council scale 0.52, 95%CI 0.40–0.64, 3 studies), and slightly greater smoking history than undiagnosed patients. Patient age, sex, current smoking status, and the presence of coughing were not associated with a previous diagnosis.

**Conclusions:**

Undiagnosed patients had less severe airflow obstruction and fewer respiratory symptoms than diagnosed patients. The lower disease burden in undiagnosed patients may significantly delay the diagnosis of COPD.

**Electronic supplementary material:**

The online version of this article (10.1186/s12931-018-0731-1) contains supplementary material, which is available to authorized users.

## Background

Chronic Obstructive Pulmonary Disease (COPD) is a lung disorder that is characterized by persistent airflow limitation [1] and associated with symptoms of shortness of breath, cough, and sputum production [2]. Patients with COPD generally seek medical attention when they experience respiratory symptoms, most notably dyspnea that is persistent and progressive [1]. However, owing to under-utilization of lung function measurements and non-specific nature of the symptoms, COPD is often not recognized until late in the disease process. Indeed, many patients do not receive a diagnosis of COPD until after being hospitalized due to a severe exacerbation [3].

Lamprecht et al. [4] reported an average underdiagnosis rate of 81% in a prevalence study that included 30,874 participants across 44 countries. Reducing risk factors such as smoking and occupational risk factors while the disease is early in its progression is an important component of treatment for COPD [5]. As such, late diagnosis of COPD represents a missed opportunity to modify the course of the disease through evidence-informed risk factor management and treatment [6, 7]. The extent of this missed opportunity is a function of the number of COPD patients who are undiagnosed, and the burden of disease (e.g., symptom burden, lung function status) in this population.

Quantifying the true burden of undiagnosed COPD and the benefit of screening and case detection can be informed by a comparative assessment of patient- and disease-factors between diagnosed and undiagnosed patients. Numerous studies have compared the characteristics of patients with undiagnosed and diagnosed COPD, but to the best of our knowledge, these studies have never been systematically compiled and pooled. We hypothesized that the characteristics of patients, their risk factors, respiratory symptoms, and disease stage influence the likelihood of receiving a diagnosis of COPD.

## Methods

### Search strategy and selection criteria

The protocol for this study is registered on the PROSPERO register of systematic reviews (CRD42017058235) [8]. We conducted a systematic review and meta-analysis to compare patient characteristics, risk factors, and symptoms in diagnosed and undiagnosed patients. We searched MEDLINE and EMBASE using the Ovid interface for eligible articles. The search strategy (Additional file 1: Appendix 1) was developed in MEDLINE and adapted to EMBASE using appropriate vocabulary terms. We included longitudinal or cross-sectional studies published in English between 1980 and April 11, 2017 that were based on original analysis of individual data. We did not assess grey literature but conference abstracts were eligible if they provided all the required information. We extracted summary data from the eligible articles and contacted the authors to obtain additional information when required (one author group provided us with additional information). Title and abstract screening were initially performed, followed by full-text analysis to determine article eligibility. We extracted data using a customized Excel spreadsheet after the eligible articles had been compiled. KJ initially performed the selection procedure, and SG independently repeated each step on a subset (10%) of articles. Discrepancies were resolved through discussions between the two reviewers. Duplicate articles found in both MEDLINE and EMBASE were identified using a reference manager and manually removed. We used the Quality Assessment Tool for Observational Cohort and Cross-Sectional Studies developed by the National Institutes of Health [9] to assign an overall quality rating (good, fair, or poor) to each study. KJ extracted relevant data and assessed the quality of the included studies, and SG replicated the assessment on 10% of articles. The reviewers determined the overall quality of each article by assigning ‘yes’, ‘no’, or ‘other’ (cannot determine, not applicable, or not reported) to 14 questions relating to external validity, bias in the measurements of the risk factors or outcomes, and confounders present in the study. The results of this assessment were interpreted qualitatively.

The population of interest in this review was adult patients (≥18 years old) with persistent airflow limitation at the time of assessment. Persistent airflow limitation was defined when the study subjects demonstrated a ratio of Forced Expiratory Volume in 1 Second (FEV_1_) to Forced Vital Capacity (FVC) < 0.7 (fixed ratio definition) [1] or FEV_1_ to FVC lower than the lower limit of normal (LLN definition) [10] after the administration of a bronchodilator during spirometry. Study subjects who had airflow limitation and also a prior diagnosis of COPD or an obstructive lung disease (emphysema, chronic bronchitis, asthma) from a health-care professional were considered to have ‘diagnosed’ COPD, whereas those with persistent airflow limitation but without a prior health professional diagnosis of COPD were considered to be ‘undiagnosed’. Studies in which COPD was not the primary disease of interest were excluded. We included studies that used either population-based (random sampling of the general population) or convenience (e.g., recruiting patients from a health-care setting) sampling.

Given the exploratory nature of the observational studies included in this review, we used a broad definition of risk factors that included any observable factor that could be associated with the probability of having received a diagnosis of COPD. Risk factors included patient-reported respiratory symptoms (cough, wheeze, phlegm, dyspnea), sex, age, current smoking status, smoking history (pack-years), and disease severity classified using the Global Initiative for chronic Obstructive Lung Disease (GOLD) grades. The relationship of interest was the association between these risk factors and the probability of having ‘diagnosed’ COPD among patients with persistent airflow limitation.

We extracted summary data from each eligible article, which included study characteristics, the definition of persistent airflow limitation that was employed in each of the studies, the method of COPD diagnosis, sampling methodology, and sample size. We also extracted the proportion or mean of risk factors between the diagnosed and undiagnosed groups, as well as the odds ratios (ORs) and their confidence intervals in studies that used regression modeling to assess the independent impact of the risk factors on diagnosis status. Studies reporting the characteristics of diagnosed and undiagnosed patients using means or proportions in contingency tables were pooled in an ‘unadjusted analysis.’ Studies that reported associations using multivariable regression modeling were pooled in a separate ‘adjusted analysis.’

### Data analysis

We used data extracted from articles measuring categorical data to generate ORs and standard errors for the association between risk factors and the probability of having received a diagnosis of COPD. In articles assessing continuous data, we calculated the mean difference (MD) in risk factors and their standard errors among diagnosed and undiagnosed patients. We pooled the ORs or MDs from individual studies using the inverse variance method implemented with the ‘meta’ package [11] in R Statistical Software [12] (version 3.3.3). We used fixed-effects models when estimates from only two studies were being pooled, or if the null hypothesis that all studies evaluated the same effect was not rejected (at 0.05 significance level) using Cochran’s Q statistic [13]. Otherwise, we used random-effects models. We quantified heterogeneity between studies using the I^2^ statistic [14]. We did not pool together studies that used alternate definitions of persistent airflow limitation (fixed ratio and LLN) given the demonstrated differences in patients meeting these criteria [15]. When separate studies used subsets of the same dataset (i.e., the Latin American Project for the Investigation of Obstructive Lung Disease [PLATINO] dataset [4, 16–18]), we used the estimate from the study with the largest sample size. We conducted a sensitivity analysis to determine the association between risk factors and COPD diagnosis only among population-based studies (as opposed to convenience sampling).

## Results

**Fig. 1:**
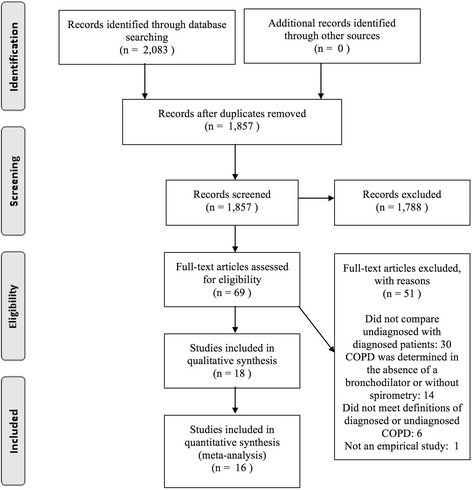
Preferred Reporting Items for Systematic Reviews and Meta-Analyses flow diagram

The search resulted in 1857 references after excluding duplicates. One thousand seven hundred eighty-eight references were excluded by screening their titles and abstracts, and 69 remained for full-text review to determine eligibility. A total of 18 articles met the inclusion criteria following the screening process, but only 16 articles were included in quantitative synthesis (Fig. 1). Two eligible articles were excluded from the meta-analysis because they were missing the necessary information [19], or did not measure any risk factors in common with other studies [20]. The overall agreement between reviewers was high (90%).

A summary of the 16 eligible articles is presented in Table 1. The majority of the 16 eligible articles were cross-sectional (*n* = 14), and population-based (*n* = 9). Other studies sampled patients from primary care clinics (*n* = 4), or among hospitalized patients (*n* = 3). Studies originated from Europe (*n* = 6), Latin America (*n* = 5), Canada (*n* = 2), and Asia (n = 1). Data from the Epidemiologic Study of COPD in Spain (EPI-SCAN) [4, 21, 22], PLATINO, and the Burden of Obstructive Lung Disease (BOLD) [4, 23], were used in three, four, and two different studies, respectively, but only one study from each dataset was included in pooled analyses. The definition of persistent airflow limitation varied between articles; 13 studies defined it as the fixed ratio, two studies used the LLN definition, and one study reported results using both definitions. Only one of the eligible articles [24] included asthma as a risk factor for assessing a previous diagnosis. The percentage of patients with undiagnosed persistent airflow limitation was greater than 50% in all but two studies (which sampled from health-care settings).

**Table 1:** Characteristics of selected studies

	Country	Study type	Population	Definition of COPD	Definition of undiagnosed COPD	Participants with COPD	Percentage undiagnosed	Quality rating
Ancochea et al. (2013) [21]	Spain	Cross-sectional *(EPI-SCAN*^a^*)*	General Population, random sample	Post-bronchodilator FEV_1_/FVC < 0.7	Spirometric obstruction and no previous diagnosis of COPD (self-reported)	386	73%	Good
Balcells et al. (2015) [3]	Spain	Prospective cohort study	Hospitalized patients, all eligible patients were invited	Post-bronchodilator FEV_1_/FVC < 0.7, 3 months after discharge	Spirometric obstruction and no diagnosis of respiratory disease or regular use of pharmacological respiratory treatment (self-reported)	342	34%	Good
Herrera et al. (2016) [25]	Argentina, Colombia, Venezuela, Uruguay	Cross-sectional	Primary care clinics, convenience sample	Post-bronchodilator FEV_1_/FVC < 0.7 and LLN	Spirometric obstruction and no previous diagnosis of chronic bronchitis, emphysema, or COPD (self-reported)	309	77%	Fair
Hill et al. (2010) [29]	Canada	Cross-sectional	Primary care clinics, convenience sample	Post-bronchodilator FEV1/FVC < 0.7 and FEV1 < 80% predicted	Spirometric obstruction and no previous diagnosis of COPD based on medical chart review over the previous 12-months	107	46%	Good
Hvidsten et al. (2010) [24]	Norway	Cross-sectional	General Population, random sample	Post-bronchodilator FEV_1_/FVC < 0.7	Spriometric obstruction and being treated by a physician or admitted to hospital for a diagnosis of obstructive lung disease (asthma, chronic bronchitis, emphysema, or COPD) in the previous 12-months (self-reported)	303	66%	Good
Labonté et al. (2016) [30]	Canada	Prospective cohort study	General Population, random sample	Post-bronchodilator FEV_1_/FVC < 0.7	Spirometric obstruction and no previous diagnosis of chronic bronchitis, emphysema, or COPD (self-reported)	505	70%	Fair
Lamprecht et al. (2015) [4]	Global	Cross-sectional *(BOLD*^b^*, PLATINO*^c^*, EPI-SCAN, PREPOCOL*^d^*)*	General Population, random sample	Post-bronchodilator FEV_1_/FVC < LLN	Spirometric obstruction and no previous diagnosis of chronic bronchitis, emphysema, or COPD (self-reported)	2992	81%	Good
Llordes et al. (2015) [31]	Spain	Cross-sectional	Primary care clinic, all eligible patients were invited	Post-bronchodilator FEV_1_/FVC < 0.7 in 2 tests 4 weeks apart (the 2nd after 4 weeks of pharmacological treatment)	Spirometric obstruction and no previous diagnosis of COPD in medical reports	422	57%	Fair
Mahishale et al. (2015) [32]	NR	Cross-sectional	Hospitalized patients, convenience sample	Post-bronchodilator FEV_1_/FVC < 0.7	Spirometric obstruction and no previous diagnosis of COPD (self-reported)	404	56%	Poor
Miravitlles et al. (2009) [22]	Spain	Cross-sectional *(EPI-SCAN)*	General Population, random sample	Post-bronchodilator FEV_1_/FVC < 0.7	Spirometric obstruction and no previous diagnosis of chronic bronchitis, emphysema, or COPD (self-reported)	408	73%	Good
Moreira et al. (2013) [16]	Brazil	Cross-sectional *(PLATINO)*	General Population, random sample	Post-bronchodilator FEV_1_/FVC < 0.7	Spirometric obstruction and no previous diagnosis of chronic bronchitis, emphysema, or COPD (self-reported)	53	62%	Fair
Nascimento et al. (2007) [17]	Brazil	Cross-sectional *(PLATINO)*	General Population, random sample	Post-bronchodilator FEV_1_/FVC < 0.7	Spirometric obstruction and no previous diagnosis of chronic bronchitis, emphysema, or COPD (self-reported)	144	88%	Fair
Queiroz et al. (2012) [27]	Brazil	Cross-sectional	Primary care clinics, convenience sample	Post-bronchodilator FEV_1_/FVC < 0.7	Spirometric obstruction and no previous diagnosis of chronic bronchitis, emphysema, or COPD (self-reported)	63	71%	Good
Schirnhofer et al. (2011) [23]	Austria	Cross-sectional *(BOLD)*	General Population, random sample	Post-bronchodilator FEV_1_/FVC < LLN	Spirometric obstruction and no previous diagnosis of chronic bronchitis, emphysema, or COPD (self-reported)	199	86%	Good
Talamo et al. (2007) [18]	Brazil, Chile, Mexico, Uruguay, Venezuela	Cross-sectional *(PLATINO*	General Population, random sample	Post-bronchodilator FEV_1_/FVC < 0.7	Spirometric obstruction and no previous diagnosis of chronic bronchitis, emphysema, or COPD (self-reported)	758	89%	Good
Zhang et al. (2013) [33]	China	Cross-sectional	Hospitalized patients, all eligible patients were invited	Post-bronchodilator FEV_1_/FVC < 0.7	Spirometric obstruction and COPD not recorded as a discharge diagnosis in medical records	705	93%	Fair

*NR* Not Reported

^a^Epidemiologic Study of COPD in Spain (EPI-SCAN)

^b^Burden of Obstructive Lung Disease (BOLD)

^c^Latin American Project for the Investigation of Obstructive Lung Disease (PLATINO)

^d^Prevalence study of COPD in Colombia (PREPOCOL)

The quality of the 16 eligible articles was variable. Nine studies were assigned a quality rating of ‘good’, six studies were assigned a rating of ‘fair’, and one was deemed poor in quality. Studies that were not assigned a ‘good’ quality rating generally had a primary study focus that was not our question of interest. The use of regression modeling to examine the independent impact of risk factors on the likelihood of receiving a COPD diagnosis was performed in seven studies, but the risk factors that were adjusted for varied substantially across studies.

### Unadjusted analysis

**Fig. 2:**
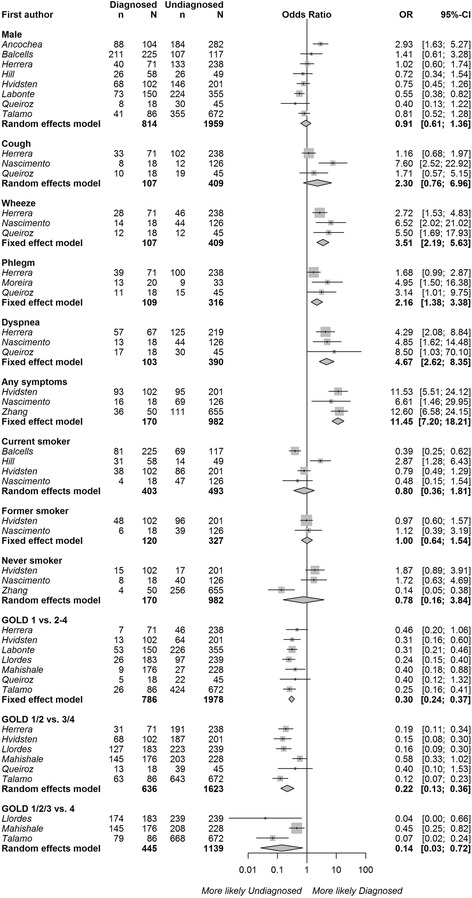
Associations between diagnosed (v. ‘undiagnosed’) COPD and sex, the presence of cough, wheeze, phlegm, dyspnea, any respiratory symptoms, smoking status, smoking history, and COPD severity based on contingency tables (‘unadjusted analysis’). Persistent airflow limitation was defined as post-bronchodilator FEV_1_/FVC < 0.7. Squares represent individual study estimates with the size of the square corresponding to their weight in the pooled estimate (represented with diamonds)

The characteristics of diagnosed and undiagnosed patients meeting the fixed ratio definition of airflow limitation were compared using contingency tables (‘unadjusted analysis’) in 12 studies. The pooled results are shown in Fig. 2. Patients with ‘diagnosed’ COPD were more likely to be experiencing respiratory symptoms such as wheezing (OR 3.51, 95% CI 2.19–5.63, 3 studies), phlegm (OR 2.16, 95% CI 1.38–3.38, 3 studies), dyspnea (OR 4.67, 95% CI 2.62–8.35, 3 studies), or any respiratory symptoms (OR 11.45 95% CI 7.20–18.21, 3 studies). They were much less likely to have mild (GOLD grade I) COPD than moderate to very severe COPD (grade II-IV) as measured by GOLD grades (OR 0.30 95% CI 0.24–0.37, 7 studies). The heterogeneity between studies was relatively low (I^2^ < 35.0% for all symptoms and COPD severity). Patient sex, current smoking status, and smoking history were not associated with a COPD diagnosis. Having a cough was also not significantly associated with diagnosis status; however, variability between the three studies measuring this risk factor was particularly high (I^2^ 77.9%).

Sensitivity analysis of only the population-based studies (excluding those that used convenience sampling) revealed very similar results (*n* = 5 studies, Additional file 1: Figure S1). Pooled analysis of two studies [23, 25] using the LLN definition of airflow limitation was consistent with the findings based on fixed ratio results (Additional file 1: Figure S2); however, cough was marginally associated with diagnosis status in this analysis (OR 1.65, 95% CI 1.02–2.66).

**Fig. 3:**
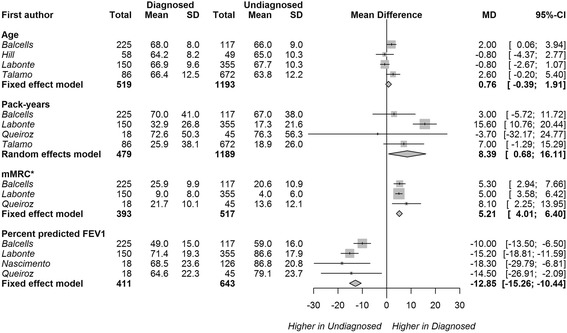
Mean difference (MD) in age, pack-years of smoking, mMRC dyspnea score, and percent of predicted FEV_1_ between diagnosed and undiagnosed categories. Persistent airflow limitation was defined as post-bronchodilator FEV_1_/FVC < 0.7. Squares represent individual study estimates with the size of the square corresponding to their weight in the pooled estimate (represented with diamonds). * modified Medical Research Council (mMRC) Dyspnea scale [26] means and standard errors (SE) for the diagnosed and undiagnosed categories are multiplied by a factor of 10

Similarly, patients with ‘diagnosed’ COPD (fixed ratio definition) were more impaired by dyspnea (modified Medical Research Council [mMRC] dyspnea scale [26] MD 0.52, 95% CI 0.40–0.64, 3 studies) and had greater airflow obstruction (percent predicted FEV_1_ MD -12.85%, 95% CI -15.26% to − 10.44%, 4 studies) than undiagnosed patients (Fig. 3). Patients with ‘diagnosed’ COPD also had a slightly greater smoking history (pack-years MD 8.39, 95% CI 0.68–16.44, 4 studies); however, there was high variability between the study means (I^2^ 84.2%). There was no difference in mean age between diagnosed and undiagnosed patients.

### Adjusted analysis

**Fig. 4:**
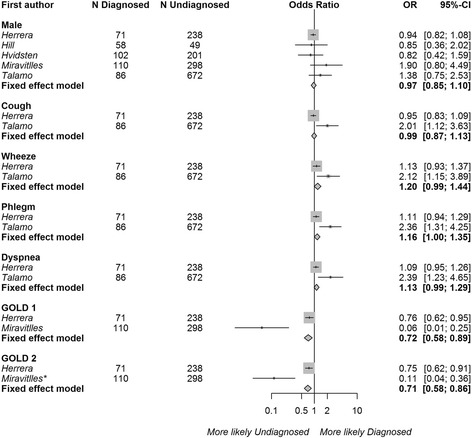
Associations between risk factors and the odds of receiving a COPD diagnosis using the regression coefficients from studies with multivariable regression modeling† (‘adjusted analysis’) and persistent airflow limitation defined as post-bronchodilator FEV_1_/FVC < 0.7. The reference categories were female, the absence of cough, wheeze, dyspnea, phlegm, and GOLD grades 3 and 4, respectively. Squares represent individual study estimates with the size of the square corresponding to their weight in the pooled estimate (represented with diamonds). †*Herrera* et al. [25] reported prevalence ratios from Poisson regression models. *The reference category was changed from GOLD grade 1 to GOLD grades 3 and 4 by assuming a covariance of 0 between the dummy variables representing GOLD grades 1 and 2.^1^ Regression models were adjusted for age (*Herrera, Hill, Hvidsten, Miravitlles, Talamo)*, sex (*Herrera, Hill, Hvidsten, Miravitlles, Talamo),* ethnicity *(Herrera, Talamo)*, body mass index *(Herrera, Hvidsten)*, education (*Herrera, Hvidsten, Miravitlles, Talamo)*, income *(Hvidsten)*, employment *(Talamo)*, risk factor to dust *(Herrera)*, smoking *(Herrera, Hill, Hvidsten, Miravitlles, Talamo)*, respiratory symptoms, *(Herrera, Hill, Hvidsten, Talamo)*, self-rated health *(Hvidsten, Miravitlles)*, COPD severity *(Herrera, Miravitlles, Talamo)*, comorbidities *(Herrera, Hvidsten)*, prior health-care use (*Herrera, Hill)*, and exacerbations *(Herrera)*

**Fig. 5:**
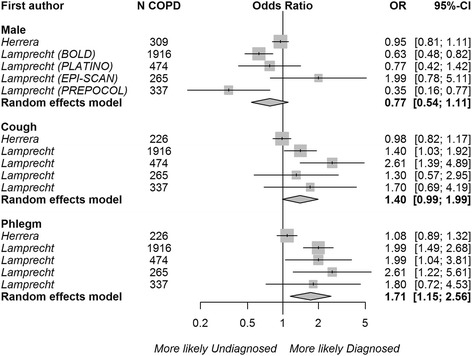
Associations between risk factors and the odds of receiving a COPD diagnosis using the regression coefficients from studies with multivariable regression modeling (‘adjusted analysis’) and persistent airflow limitation defined as post-bronchodilator FEV_1_/FVC < LLN. The reference categories were female, and the absence of cough and phlegm, respectively. The results for each dataset (BOLD, PLATINO, EPI-SCAN, PREPOCOL) analyzed in Lamprecht et al. [4] were pooled separately. Squares represent individual study estimates with the size of the square corresponding to their weight in the pooled estimate (represented with diamonds).^1^ Regression models were adjusted for age (*Herrera, Lamprecht)*, sex *(Herrera, Lamprecht)*, ethnicity *(Herrera)*, body mass index *(Herrera)*, education *(Herrera, Lamprecht)*, risk factors to dust *(Herrera)*, smoking *(Herrera, Lamprecht)*, respiratory symptoms (*Herrera, Lamprecht)*, COPD severity *(Herrera, Lamprecht),* comorbidities *(Herrera)*, and prior health-care use *(Herrera, Lamprecht)*

Articles using regression modeling to assess the independent impact of risk factors on COPD diagnosis (‘adjusted analysis’) were pooled by risk factor type, and the results are presented in Fig. 4 for the fixed ratio definition of persistent airflow limitation (5 articles), and Fig. 5 for the LLN definition (2 articles with 5 datasets). Compared with the unadjusted analysis, the effect sizes of the risk factors were attenuated in these analyses. The presence of phlegm had a weak independent association with the diagnosis of COPD (OR 1.16, 95% CI 1.00–1.35, 2 studies) using the fixed ratio definition. The presence of wheezing (OR 1.20, 95% CI 0.99–1.44, 2 studies) and dyspnea (OR 1.13 95% CI 0.99–1.29, 2 studies) showed a trend towards association. Mild COPD (GOLD grade I OR 0.72, 95% CI 0.58–0.80) or moderate COPD (GOLD grade II, OR 0.71, 95% CI 0.58–0.86) were independently associated with a lower likelihood of diagnosis, compared with severe or very severe (reference GOLD grades III-IV). Sex and the presence of cough did not influence the likelihood of being diagnosed in the adjusted analyses. Overall, heterogeneity in the effect estimates between studies was very high (I^2^ > 70.0% for all risk factors except sex).

Three risk factors were pooled in our assessment of studies using adjusted analysis based on the LLN definition of persistent airflow limitation. This analysis indicated a more strongly positive association between the presence of phlegm and being diagnosed with COPD (OR 1.71, 95% CI 1.15–2.56), although there was heterogeneity between datasets (I^2^ 75.2%). Patient sex and the presence of cough had no independent effects.

## Discussion

The presence of respiratory symptoms and GOLD grade III or IV disease severity were strongly associated with a prior diagnosis of COPD among individuals with persistent airflow limitation on spirometry. These findings were relatively consistent across analysis methods and alternate definitions of persistent airflow limitation. Greater disease severity was the most important characteristic of diagnosed patients in two out of three pooled analyses. In particular, patients with mild or moderate COPD (as measured by GOLD grades) were 78% less likely to have received a diagnosis than patients with severe or very severe COPD in the unadjusted analysis (based on contingency tables), and mean percent predicted FEV_1_ was 13% lower in diagnosed than undiagnosed patients. Disease severity was also the only risk factor that was associated with a diagnosis in both the unadjusted and adjusted (based on regression modeling) analyses. In the adjusted analysis, patients with moderate COPD were 29% less likely to have received a diagnosis than patients with severe or very severe COPD.

Among respiratory symptoms, the presence of dyspnea was the most strongly associated with a previous diagnosis in the unadjusted analysis. Patients with ‘diagnosed’ COPD scored 0.52 points higher on the mMRC dyspnea scale. One study [27] provided evidence that the mean score on the mMRC scale could have been used to distinguish undiagnosed from diagnosed patients using commonly accepted criteria (‘more dyspnea’ if mMRC score ≥ 2 v. ‘less dyspnea’ if mMRC score < 2) [1]. Following dyspnea, the presence of wheeze and phlegm were also strongly associated with ‘diagnosed’ COPD in the unadjusted analysis. These associations were weaker in the adjusted analysis but were still present. Interestingly, the presence of coughing was not well associated with a previous diagnosis in any of the pooled analyses. Overall, aside from the attenuated results in the adjusted analysis (discussed in detail below), our findings suggest a strong association between the presence of dyspnea, phlegm, or wheeze and a COPD diagnosis. This association is likely because patients with respiratory symptoms are more likely to seek care, and doctors are more likely to test for COPD in patients with symptoms [1].

Overall, these findings suggest that a diagnosis of COPD is likely to be delayed in patients with a lower burden of respiratory symptoms and lung function decline that progresses more slowly, meaning that opportunities for early intervention may be lower in these patients. Patients with fewer respiratory symptoms are also less likely to be screened for COPD following the U.S. Preventive Services Task Force recommendation [28]. However, our results suggest that screening or case detection methods that rely exclusively on the presence of respiratory symptoms may be missing undiagnosed patients. Symptoms should be assessed in combination with a history of exposure to risk factors for COPD when considering a diagnosis [1].

Importantly, sex, age, and smoking status were not independently associated with receiving a diagnosis of COPD in any of the pooled analyses. Because these are major risk factors for the presence of COPD [1], physicians should be more likely to diagnose COPD in older patients who are current or past smokers, which should have resulted in an independent association between these factors and a diagnosis of COPD. The lack of such association in any of our analyses may indicate that the relation between these risk factors and the presence of COPD is not sufficiently recognized by physicians. This is in contrast to the strong association of GOLD grade and symptoms with diagnosis status, which suggests that a diagnosis of COPD is more commonly made based on the degree of impairment in lung function and/or patient respiratory symptoms. This hypothesis might also explain our finding that smoking history in terms of pack-years was associated with a previous diagnosis of COPD while current smoking status was not, as only smoking history is related to cumulative lung function impairment.

The effects of risk factors on the likelihood of being diagnosed were weaker in the adjusted analyses than in the unadjusted analyses. The adjusted analyses were based on pooled coefficients from regression modeling. Although the inclusion of covariates is expected to reduce the effects sizes compared to odds ratios derived from contingency tables (as in the unadjusted analysis), one study in the adjusted analysis [25] had unusual results that received disproportionate weighting. In contrast to all other studies in this review, Herrera et al. [25] found that respiratory symptoms were not associated with the likelihood of having received a diagnosis of COPD. In the adjusted analysis, these results were pooled with one other study [18], which found that the presence of respiratory symptoms were strongly associated with the likelihood of receiving a diagnosis. This discrepancy between studies may be due to differences in the population that was sampled (primary care clinic [25] versus general population [18]). In general, studies in clinic settings might have observed smaller differences between undiagnosed and diagnosed patients because they sampled from a subset of patients that were prompted to seek care because of symptoms.

Our systematic review has several strengths. First, we used data from a total of 16 articles in the meta-analysis, and these articles were mostly population-based studies that scored high in quality. Second, there were a robust number of studies for many risk factors; patient sex was assessed in 10 studies in total, followed by disease severity in 9 studies, and respiratory symptoms and smoking history in 8 studies. The methods used to measure disease severity, respiratory symptoms, and smoking history were relatively consistent across studies, which facilitated pooling of their findings. Lastly, we conducted several pooled analyses to assess the sensitivity of our findings to alternate definitions of COPD (fixed ratio and LLN) and analysis methods (unadjusted and adjusted analyses). Except for one study [25], our findings were consistent.

Our systematic review also has several limitations. First, half of the pooled samples were based on data from three large prevalence studies (EPI-SCAN, PLATINO, and BOLD). This resulted in overrepresentation of patients in Spain and Latin America; differences in patient and physician behavior and health-care services use can result in findings that vary across settings. Second, although the total number of studies for each risk factor was robust, the number of studies assessing each risk factor within pooled analyses tended to be small. This was partly because separate articles using the same dataset could not be combined in our pooled analyses. Third, with the exception of dyspnea, all other respiratory symptoms in the pooled analyses were measured as binary variables (either present or absent). Given our finding that symptoms are characteristic of a COPD diagnosis, a more nuanced assessment of their severity might result in an even greater ability to distinguish between undiagnosed and diagnosed patients. In addition, because respiratory symptoms were self-reported in all studies, reporting bias might have exaggerated the difference in symptoms between the undiagnosed and diagnosed groups. Finally, several studies reported additional comparisons of risk factors between diagnosed and undiagnosed patients. Examples include education, income, comorbidities, quantity and type of care, and a previous diagnosis of asthma. However, due to inconsistent definitions and different categorizations, we could not pool these estimates across studies. An important knowledge gap is the impact of environmental risk factors on the likelihood of receiving a diagnosis, which was rarely measured in the included studies.

## Conclusions

The findings from this systematic review have important implications for research and policy around COPD diagnosis, for example, in estimating the return on investment in screening and case detection strategies for COPD. The true burden of COPD is the sum of the disease burden in diagnosed and undiagnosed patients. Our results indicate that undiagnosed patients generally have milder disease and therefore a lower disease burden, meaning that strategies aiming to reduce the underdiagnosis problem are unlikely to result in immediate and dramatic improvements in patient-related outcomes such as symptoms. However, the gap in disease severity and symptom burden between diagnosed and undiagnosed patients also indicates a delay in COPD diagnosis. Given the potential for disease modification at early stages of COPD, reducing this delay could be associated with substantial improvement in long-term patient outcomes and a reduction in mortality and costs.

## Additional file

Additional file 1: Supplementary Material. (DOCX 830 kb)

## Abbreviations

BOLD: Burden of Obstructive Lung Disease
COPD: Chronic Obstructive Pulmonary Disease
EPI-SCAN: Epidemiologic Study of COPD in Spain
FEV_1_: Forced Expiratory Volume in 1 s
FVC: Forced Vital Capacity
GOLD: Global Initiative for chronic Obstructive Lung Disease
MD: Mean difference
mMRC: modified Medical Research Council
OR: Odds ratio
PLATINO: Latin American Project for the Investigation of Obstructive Lung Disease
PREPOCOL: Prevalence study of COPD in Colombia
