# Supplementary material for: Characterizing undiagnosed chronic obstructive pulmonary disease: a systematic review and meta-analysis
Source: Respir Res. 2018 Feb 7;19:26. doi: 10.1186/s12931-018-0731-1 (PMC5803996; doi:10.1186/s12931-018-0731-1)
Supplement: Supplementary file 1 — Supplementary Material. (DOCX 830 kb) [file 12931_2018_731_MOESM1_ESM.docx]

## ADDITIONAL FILE 1

**Appendix 1:** Search strategy

**Figure S1:** Associations between diagnosed (v. ‘undiagnosed’) COPD and sex, the presence of any respiratory symptoms, smoking status, smoking history, and COPD severity based on the contingency tables of studies using random sampling of the general population (‘unadjusted analysis’). Persistent airflow limitation was defined as post-bronchodilator FEV_1_/FVC<0·7. Squares represent individual study estimates with the size of the square corresponding to their weight in the pooled estimate (represented with diamonds).

**Figure S2:** Associations between diagnosed (v. ‘undiagnosed’) COPD and sex, the presence of cough, wheeze, phlegm, dyspnoea, and COPD severity based on contingency tables (‘unadjusted analysis’). Persistent airflow limitation was defined as post-bronchodilator FEV_1_/FVC< lower limit of normal (LLN). Squares represent individual study estimates with the size of the square corresponding to their weight in the pooled estimate (represented with diamonds).

**Appendix 1: Search strategy**

**MEDLINE (OVID)**

March 22, 2017

Database: Ovid MEDLINE(R) In-Process & Other Non-Indexed Citations and Ovid MEDLINE(R) <1946 to Present>

Search Strategy:

--------------------------------------------------------------------------------

1 *pulmonary disease, chronic obstructive/ or *bronchitis, chronic/ or *pulmonary emphysema/ (38797)

2 *airway obstruction/ (11509)

3 *bronchitis/ or *bronchiolitis/ or *bronchiolitis obliterans/ or *cryptogenic organizing pneumonia/ (18102)

4 *emphysema/ or *mediastinal emphysema/ or *subcutaneous emphysema/ or *alpha 1-antitrypsin deficiency/ (11041)

5 *Lung Diseases, Obstructive/ (13636)

6 limit 5 to yr="1980 -2001" (11016)

7 or/1-4,6 (84739)

8 di.fs. [Diagnosis] (2370855)

9 ep.fs. [Epidemiology] (1471514)

10 8 or 9 (3570350)

11 7 and 10 (23089)

12 Diagnostic Errors/ (35551)

13 Delayed Diagnosis/ (4539)

14 Early Diagnosis/ (22251)

15 Airway Obstruction/di [Diagnosis] (2772)

16 underdiagnos$.mp. (7477)

17 under diagnos$.mp. (2788)

18 undiagnos$.mp. (16208)

19 "Diagnostic Techniques and Procedures"/ (2914)

20 Diagnosis, Differential/ (432511)

21 "not diagnos$".mp. (5636)

22 misdiagnos$.mp. (26521)

23 or/12-22 (533328)

24 11 and 23 (4893)

25 "Risk Factors"/ (717586)

26 logistic models/ (119410)

27 risk assessment/ (224203)

28 risk factors/ (717586)

29 risk/ (115068)

30 protective factors/ (1844)

31 probability/ (54508)

32 odds ratio/ (79810)

33 risk factor$.mp. (954945)

34 risk assessment$.mp. (251708)

35 (characteri#tic? or characteri#e? or characteri#ation).mp. (2499735)

36 or/25-35 (3674000)

37 24 and 36 (992)

38 limit 37 to yr=1980 -current (981)

**EMBASE (OVID)**

April 11, 2017

Database: Embase <1974 to 2017 August 14>

Search Strategy:

--------------------------------------------------------------------------------

1 *chronic obstructive lung disease/ or *chronic bronchitis/ or *lung emphysema/ (68276)

2 *airway obstruction/ or *airflow limitation/ (10588)

3 *bronchitis/ or *chronic bronchitis/ or *bronchiolitis/ or *bronchiolitis obliterans/ or *bronchiolitis obliterans organizing pneumonia/ (24818)

4 *emphysema/ or *subcutaneous emphysema/ or *cigarette smoke-induced emphysema/ or *elastase-induced emphysema/ or *experimental emphysema/ or *alpha 1 antitrypsin deficiency/ (9667)

5 or/1-4 (103405)

6 di.fs. [Diagnosis] (2921008)

7 ep.fs. [Epidemiology] (976523)

8 6 or 7 (3661177)

9 5 and 8 (21021)

10 Diagnostic Error/ (50975)

11 Early Diagnosis/ (89985)

12 *Airway Obstruction/di [Diagnosis] (1452)

13 Diagnostic Procedure/ (80939)

14 Differential diagnosis/ (334345)

15 or/10-14 (535842)

16 9 and 15 (3600)

17 Risk Factor/ (828169)

18 regression analysis/ (116928)

19 multivariate analysis/ (141771)

20 risk assessment/ (424757)

21 risk factor/ (828169)

22 risk/ (496728)

23 probability/ (75672)

24 odds ratio/ (12341)

25 risk factor$.mp. (1080390)

26 risk assessment$.mp. (444895)

27 (characteri#tic? or characteri#e? or characteri#ation).mp. (3007687)

28 or/17-27 (4767936)

29 16 and 28 (717)

30 underdiagnos$.mp. (10792)

31 under diagnos$.mp. (5242)

32 undiagnos$.mp. (23402)

33 "not diagnos$".mp. (8488)

34 misdiagnos$.mp. (37341)

35 unrecogni$.mp. (33790)

36 Delayed Diagnosis/ (9134)

37 or/30-36 (123603)

38 9 and 37 (663)

39 29 or 38 (1299)

40 limit 39 to yr="1980 -current" (1296)

41 limit 40 to "english language" (1049)

42 40 not 41 (247)


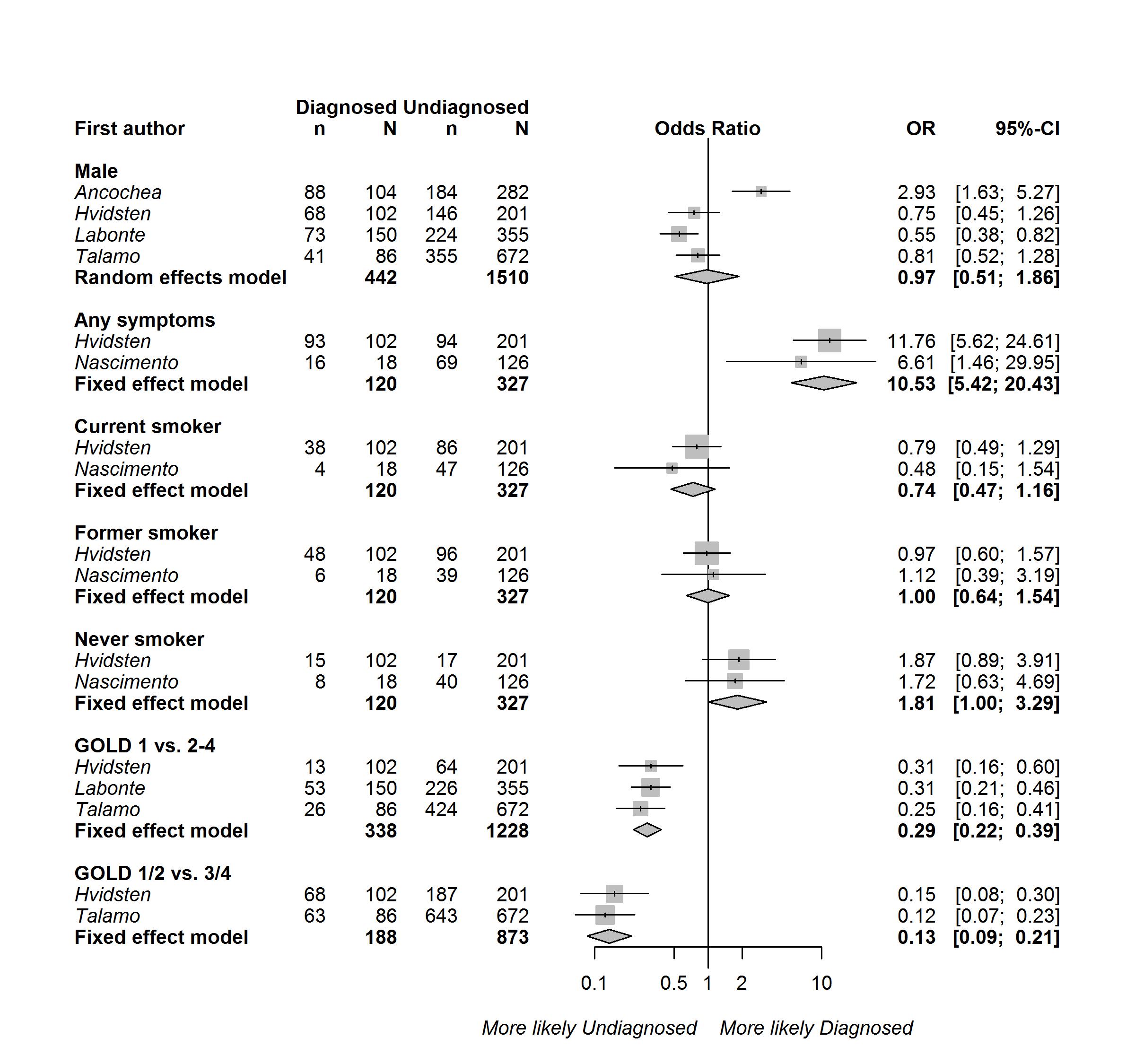


**Figure S1:** Associations between diagnosed (v. ‘undiagnosed’) COPD and sex, the presence of any respiratory symptoms, smoking status, smoking history, and COPD severity based on the contingency tables of studies using random sampling of the general population (‘unadjusted analysis’). Persistent airflow limitation was defined as post-bronchodilator FEV_1_/FVC<0.7. Squares represent individual study estimates with the size of the square corresponding to their weight in the pooled estimate (represented with diamonds).

###
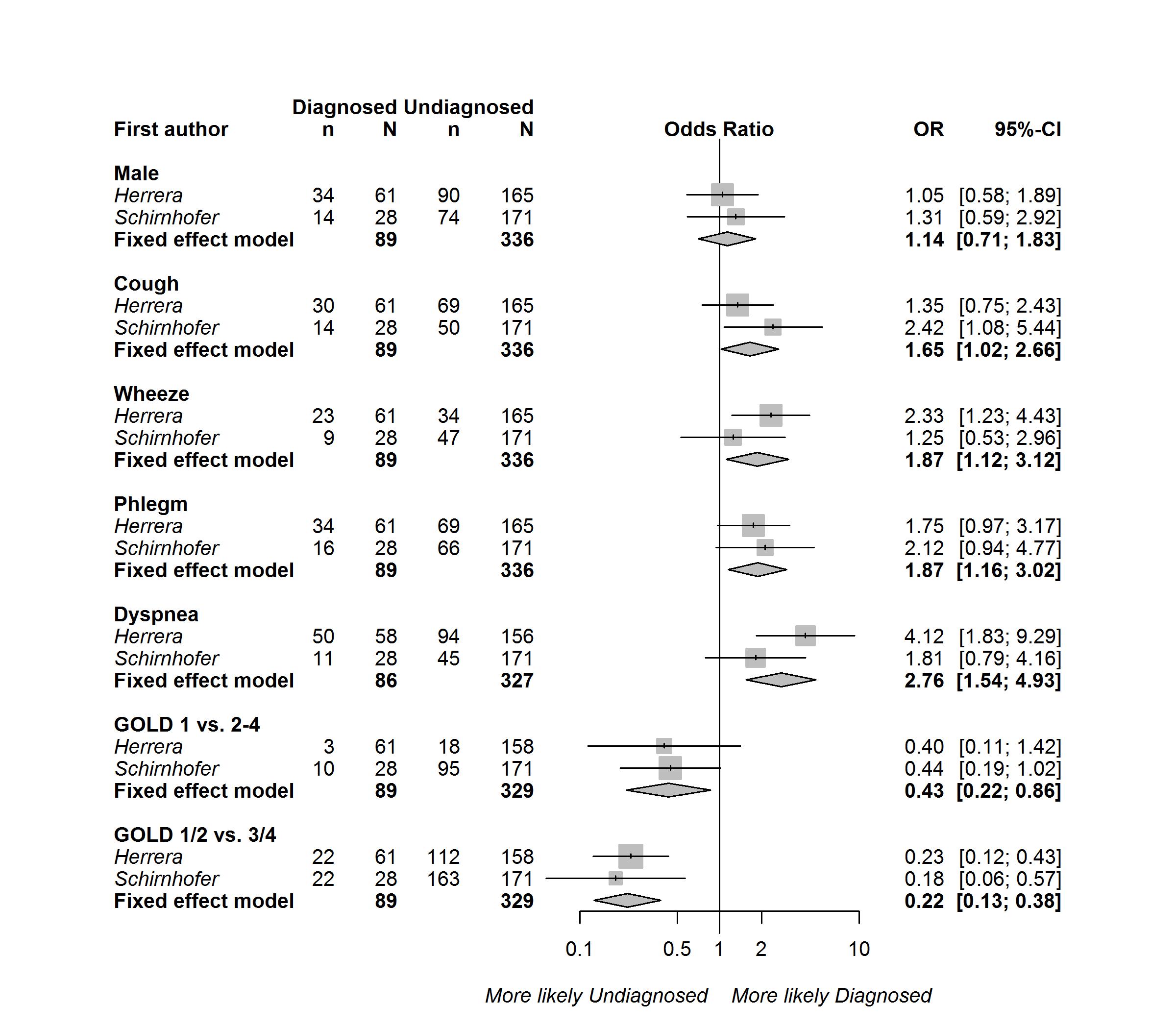


**Figure S2:** Associations between diagnosed (v. ‘undiagnosed’) COPD and sex, the presence of cough, wheeze, phlegm, dyspnoea, and COPD severity based on contingency tables (‘unadjusted analysis’). Persistent airflow limitation was defined as post-bronchodilator FEV_1_/FVC< lower limit of normal (LLN). Squares represent individual study estimates with the size of the square corresponding to their weight in the pooled estimate (represented with diamonds).
